# Maternal dietary iron intake during pregnancy has a potential effect on the neonate gut microbiota profile

**DOI:** 10.3389/fnut.2025.1589258

**Published:** 2025-06-24

**Authors:** Qi Qi, Danmeng Liu, Liang Wang, Yingze Zhu, Mitslal Abrha Gebremedhin, Zhonghai Zhu, Lingxia Zeng

**Affiliations:** ^1^Department of Epidemiology and Biostatistics, School of Public Health, Xi’an Jiaotong University Health Science Center, Xi’an, China; ^2^Translational Medicine Center, Northwest Women’s and Children’s Hospital, Xi’an, Shaanxi, China; ^3^Center for Chronic Disease Control and Prevention, Global Health Institution, Xi’an Jiaotong University, Xi’an, China; ^4^Key Laboratory for Disease Prevention and Control and Health Promotion of Shaanxi Province, Xi’an, China; ^5^Nutrition and Food Safety Engineering Research Center of Shaanxi Province, Xi’an, China

**Keywords:** dietary iron, pregnancy, mother, neonate, gut microbiota

## Abstract

**Introduction:**

Iron is an essential nutrient during pregnancy and may influence the early development of the neonatal gut microbiota. This study aimed to investigate the association between maternal dietary iron intake during pregnancy and the gut microbiota (GM) characteristics of both the mother and neonate in a well-characterized cohort.

**Methods:**

Ninety-five mother-neonate dyads were included in this study. Mothers completed a food frequency questionnaire (FFQ) providing estimates of dietary iron intake during pregnancy, and participants were categorized into higher (≥ median) or lower (< median) groups of maternal dietary iron intake. Fecal samples were collected from mothers (third trimester) and from neonates, and assessed via 16S rRNA amplicon sequencing. Differences in diversity and abundance of GM were compared between groups.

**Results:**

There was no difference in profile or diversity in maternal samples however, neonatal samples indicated greater diversity of GM in infants of mothers with higher intakes of iron (Shannon *p* = 0.04; Simpson *p* = 0.01). After stratification by delivery mode, in the stratum of normal vaginal delivery (NVD), Simpson diversity remained higher in the infants’ GM of mothers with higher intakes of iron (*p* = 0.04). The relative abundance of the core genus *Bifidobacterium* in NVD and cesarean section (CS) neonates showed higher in the higher group than that in the lower group, as the difference was not statistically significant. Maternal dietary iron intake was significantly associated with the neonate GM composition with variation explained 10.24% (*p* = 0.007).

**Conclusion:**

Adequate dietary iron intake during pregnancy may promote beneficial bacterial colonization and increase the biodiversity of the neonate GM.

## 1 Introduction

Iron stands as an essential regulator in shaping the gut microbiota, influencing both its composition and function in multifaceted ways (1–3). At the cellular level, iron serves as a critical cofactor involved in catalyzing several microbial enzymes pivotal for cellular processes and metabolic pathways (4, 5). Thus, the interaction between iron and the gut microbiota reveals a potential association that significantly influences the delicate balance of the microbial ecosystem.

Recent evidence suggests that maternal dietary iron intake during pregnancy may impact the development of the maternal and infant GM (6, 7). Consuming excess dietary iron or iron supplements has the potential to increase pathogenic bacteria at the expense of beneficial bacteria, and modify the composition and structure of the GM, especially in infants (8, 9). However, the relationship between maternal dietary iron intake and the characteristics of the mother and neonate GM remains poorly understood.

This study aims to investigate the association between maternal dietary iron intake and characteristics of the GM in mother-neonate dyads in a well characterized cohort.

## 2 Materials and methods

### 2.1 Study design and participant enrolment

The study collected stools samples and food frequency questionnaires during pregnancy from 95 mother-neonate dyads between January 2018 and June 2019 (10, 11). Inclusion criteria were: (1) Full-term singleton pregnancies; (2) detailed dietary records for the period of gestation and; (3) matched information and fecal samples from mother-neonate dyads. Participants were excluded if there were (1) gestational complications; (2) incomplete or poor-quality stool samples and diet histories; (3) the mother that received antibiotics within 7 days before FFQ. Supplementary Figure 1 describes the process of enrolment.

### 2.2 Sociodemographic information

A face-to-face questionnaire was used to collect information from the mothers during their antenatal care (ANC) visits. In the initial and following ANC visits, data about the maternal socio-demographic characteristics, reproductive history and details regarding the pregnancy were documented. Birth outcomes were retrieved from the hospital information system.

### 2.3 Maternal dietary iron intake

A 107-item semi-quantitative food frequency questionnaire (FFQ) with good reliability and validity for pregnant women was used in this study (12). The FFQ was developed based on a previously validated FFQ utilized for pregnant women in Shaanxi (13, 14). A 24-h dietary recall (an interviewer-administered method in which participants report all foods and beverages consumed in the previous 24 h) was collected from mothers and iron intakes from these compared to those of the FFQ. A correlation coefficient of 0.65 was calculated, based on the Chinese Food Composition Tables (15).

Previous research indicates that maternal dietary intake pattern in Shaanxi is relatively stable during pregnancy (16–18), therefore, a FFQ taken upon admission just prior to delivery or within three days post-delivery was considered a reliable indicator of average iron intake per day for the duration of the pregnancy. Average macro and micronutrient intake was calculated using the Chinese Food Composition Table (19), adjusted for differences in energy intake and analyzed using the regression residual analysis (20). Pregnant women in the investigation region generally have a lower daily iron intake than intake recommended by WHO (30–60 mg/during pregnancy) (21), and by Chinese Nutrition Association (24 mg/d during mid-pregnancy) (22). In order to better illustrate the effect, participants were divided into two groups as Higher iron (≥ median) and Lower iron (< median) based on intakes either higher or lower than the group median rather than using the recommended values as a cut-off.

### 2.4 Fecal sample collection, DNA extraction and high-throughput 16S rRNA gene amplicon sequencing

Fecal samples were collected from mothers in hospital prior to delivery, while fecal samples from neonates were collected from diapers within three days of delivery. All the samples were stored temporarily in a −20°C refrigerator and then stored in a −80°C refrigerator for later experiments. Bacterial DNA was extracted using the QIAamp Fast DNA stool Mini Kit (Qiagen). PCR amplification targeted the variable regions V3–V4 of the 16S rRNA gene. Extracted DNA was sequenced using the Hiseq 2500 platform (Biomarker Technologies Co., Ltd, Beijing, China). Data were stored in FASTQ format files and annotation files for taxa of interest were applied using the reference database Silva 16S rRNA version 115 (23), batch analysis were conducted with satisfaction and then, amplicon sequence variants (ASVs) were inferred after quality control.

### 2.5 Bioinformatic and statistical analysis

The Phyloseq package (24) was used to create objects for the measurement of diversity indices and microbiota analysis in R. In order to maximally retain the diversity of fecal samples, we kept the sequencing depth of each sample without rarefaction (25). In addition, considering the low biomass in neonate fecal samples and potentially spurious taxa due to sequencing errors, we filtered the ASV data using the filter_taxa function, and only ASVs present at least 2 counts in at least 10% of fecal samples (e.g., based on our sample size, ASVs present in at least 15 fecal samples) were retained. The microbiota characteristics of fecal samples were compared between the maternal dietary iron intake groups, then further stratified by delivery mode for analyses to reduce differences in colonization patterns due to delivery mode (26). Alpha diversity indices including Chao1, Shannon, and Simpson diversity were calculated via filtered ASV table using the microbiota package (27), then group differences were compared by Mann–Whitney U test. To identify the effect of maternal dietary iron intake on the maternal/neonatal GM community, permutation multivariate analysis of variance (PERMANOVA) with 9999 permutations was conducted using (1) the adonis2 function in the vegan package and visualized via Principal Coordinate Analysis (PCoA) using the vegan (28) and ggplot2 (29) packages; and then (2) stratified by delivery mode and adjusting for the effect of potential confounding variables such as pre-pregnancy BMI, and iron supplementation during pregnancy. Moreover, GM composition was compared at phylum level, with top phyla visualized using the plot_composition function. Neonate fecal samples were then stratified by delivery mode compared at genus level with top genera.

The shared core ASVs among groups in maternal and neonate fecal samples were illustrated using Venn diagrams. Additionally, Canonical correspondence analysis (CCA) was employed to assess the correspondence between maternal dietary iron intake as independent variables of host environmental factors and the composition of the neonate microbiota at the genus level. Finally, differences in taxonomic abundance were assessed between two groups stratified by delivery mode, performed using Analysis of Compositions of Microbiomes with Bias Correction (ANCOM-BC) (v1.2.2) (30), with adjustments made for neonate feeding, maternal use of Intrapartum Antimicrobial Prophylaxis (IAP), iron supplementation during pregnancy, and batch effect. Correction values obtained from the models were adjusted via the Bonferroni method (*q* < 0.05). Taxa with a proportion of zeroes greater than 90% were excluded.

## 3 Result

### 3.1 Demographic characteristics and maternal dietary iron intake

Among the 95 mother-neonate dyads, 73.7% of neonates were delivered vaginally. The demographic characteristics of the mothers and neonates are summarized in Table 1. The maternal dietary iron intake was determined with a median (P25, P75) of 19.4 (16.9, 21) mg/d. There were no statistically significant differences in socio-demographic characteristics between the groups, except for maternal dietary iron intake, which was significantly different in both the NVD and CS groups (*p* < 0.001, respectively).

**TABLE 1:** Baseline characteristics of mother and neonate grouped by maternal dietary iron intake (mg/d) during pregnancy.

Variables	General	NVD (*n* = 70)		CS (*n* = 25)	
		Higher iron	Lower iron	Higher iron	Lower iron
*N*	95	34	36	12	13
**Mother**					
**Maternal age, *n* (%)**					
< 25	36 (37.9)	13 (36.1)	14 (38.9)	4 (11.1)	5 (13.9)
≥ 25	59 (62.1)	21 (35.6)	22 (37.3)	8 (13.6)	8 (13.6)
**Education years, *n* (%)**					
< 9	56 (59.0)	20 (35.7)	21 (37.5)	9 (16.1)	6 (10.7)
≥ 9	39 (41.1)	14 (35.9)	15 (38.5)	3 (7.7)	7 (18.0)
**Occupation, *n* (%)**					
Farmer	77 (81.1)	24 (31.2)	32 (41.6)	10 (13.0)	11 (14.3)
Non-farmer	18 (18.9)	10 (55.6)	4 (22.2)	2 (11.1)	2 (11.1)
Maternal height (cm),mean ± SD	160 ± 5	160 ± 5	160 ± 5	160 ± 4	160 ± 3
Maternal weight (kg),mean ± SD	54.2 ± 7.7	53.8 ± 6.4	53.7 ± 8.2	54.8 ± 7.4	56.3 ± 9.9
Pre-pregnancy BMI (kg/m^2^),mean ± SD	21.3 ± 2.6	21.2 ± 2.6	20.9 ± 3.1	21.6 ± 2.8	22.1 ± 3.3
**Neonate**					
**Sex, *n* (%)**					
Male	47 (49.5)	14 (29.8)	18 (38.3)	6 (12.8)	9 (19.2)
Female	48 (50.5)	20 (41.7)	18 (37.5)	6 (12.5)	4 (8.3)
Gestational age (week)^a^, median (P25–P75)	39.2 (39.1–40.1)	39.2 (39–40.2)	39.2 (39.1–40.1)	39.2 (39.1–40.6)	40.1 (39.2–40.1)
Birth weight (g)^a^, mean ± SD	3,247 ± 406	3,233.5 ± 466.3	3,188 ± 279.9	3,345.8 ± 439.8	3,350 ± 498.3
**Maternal dietary iron intake (mg/d)**					
Median (P25–P75)	19.4 (16.9–21.0)	20.8 (20.0–22.3)	17.6 (15.9–18.7)	21.6 (20.6–22.7)	16.9 (16.6–18.7)
Min–Max	10.1–30.6	19.5–30.6	10.1–19.4	19.7–29.3	13.7–19.5

*^a^*data missing (*n* = 1). Maternal dietary iron intake was significantly higher in the higher group than that in the lower group in both NVD and CS delivery mode (*p* < 0.001, respectively). NVD, normal vaginal delivery; CS, cesarean section.

### 3.2 Overview of maternal and neonate GM composition

The microbiota distribution in groups is shown in Supplementary Figures 2, 3. Maternal GM were dominated by Firmicutes [mean (%) ± SD (%), 0.61 ± 0.2], and there was no significant difference between maternal higher iron group and lower iron group. While in neonates of NVD, Proteobacteria was the dominant phylum [mean (%) ± SD (%), 0.41 ± 0.32], followed by Firmicutes [mean (%) ± SD (%), 0.3 ± 0.25] in the neonate GM, and in CS neonates, Firmicutes [mean (%) ± SD (%), 0.44 ± 0.28] followed by Proteobacteria [mean (%) ± SD (%), 0.33 ± 0.31] dominated. No significant difference was found at phylum and genus level in the microbiota relative abundance between maternal higher and lower iron group (*p* > 0.05, respectively).

### 3.3 Maternal dietary iron intake and diversity of the GM

**FIGURE 1:**
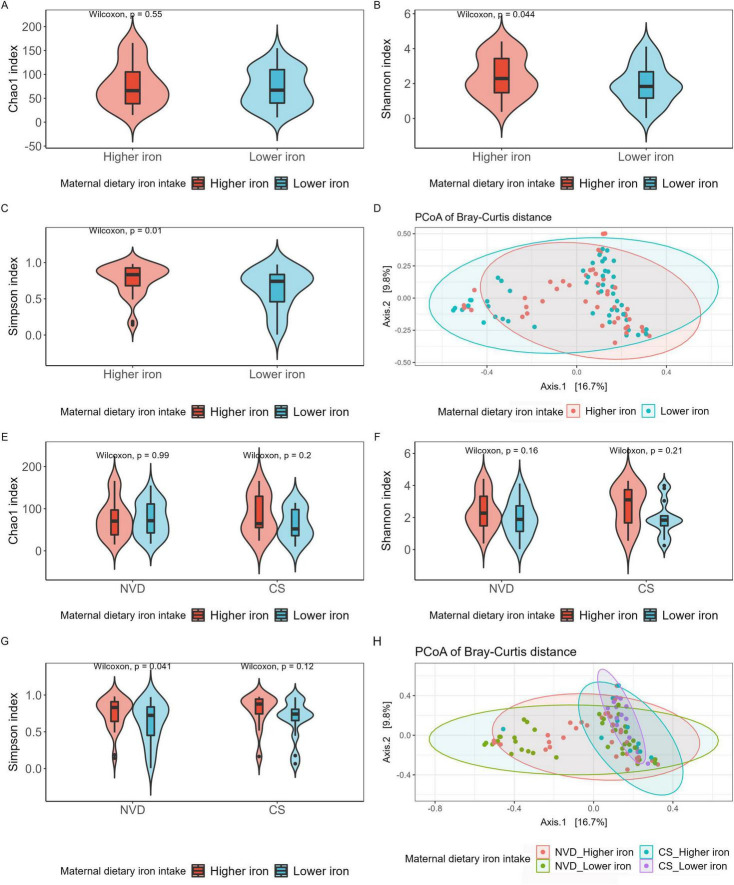
Alpha and beta diversities of neonate gut microbiota grouped by maternal dietary iron intake into Higher iron and Lower iron groups **(A–D)**, then stratified by delivery mode and grouped by Higher iron and Lower iron **(E–H)**. **(A)** Chao1 index regarding the microbial community richness of the neonate gut microbiota; Shannon **(B)** (Higher iron vs. Lower iron, *p* = 0.044) and Simpson **(C)** (Higher iron vs. Lower iron, *p* = 0.01) index regarding the microbial community diversity of the neonate gut microbiota; **(D)** PCoA analysis of bray_curtis distance regarding the difference in the microbial community composition (PERMANOVA with 9999 permutations, *p* > 0.05). **(E)** Chao1 index regarding the microbial community richness of the neonate gut microbiota; Shannon **(F)** and Simpson **(G)** (Higher iron vs. Lower iron in VD, *p* = 0.041) index regarding the microbial community diversity of the neonate gut microbiota; Each box plot represents the median, interquartile range, minimum, and maximum values. **(H)** PCoA analysis of bray_curtis distance regarding the difference in the microbial community composition (PERMANOVA with 9999 permutations, *p* > 0.05).

The alpha and beta diversity of mother fecal samples showed no significant difference between lower and higher dietary iron intake groups (Supplementary Figure 4). However, the Shannon and Simpson indices in the higher iron group were significantly higher than that in the lower iron group for neonate fecal samples (Shannon index, *p* = 0.044; Simpson index, *p* = 0.010; Figures 1B, C), with no significant difference in Chao1 index (Figure 1A). Meanwhile, after stratification, the results showed the same tendency regarding Simpson diversity between the higher and lower maternal dietary iron intake groups in NVD neonates (*p* = 0.041; Figure 1G), and no significant difference was observed for Chao1 and Shannon diversity (Figures 1E, F). Furthermore, beta diversity presented no significant difference in neonate microbial community structure among two and the four groups (Figures 1D, H, *p* > 0.05).

### 3.4 Core genera and shared ASVs in mother-neonate dyads

**FIGURE 2:**
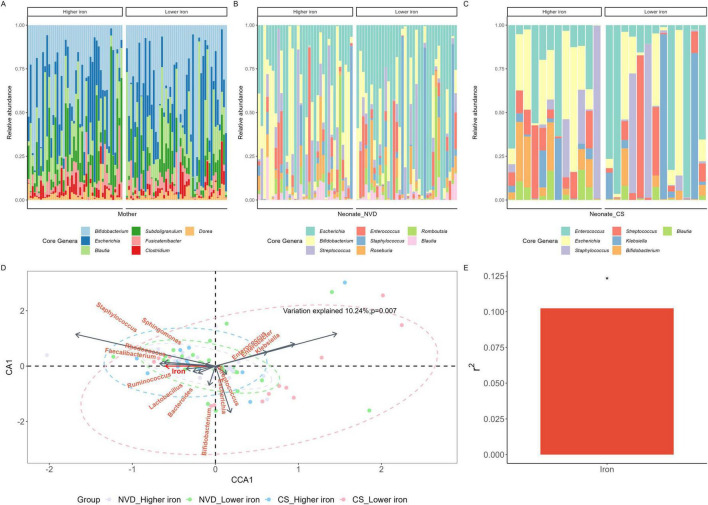
Core genus of the mother **(A)**, NVD neonate **(B)** and CS neonate **(C)** samples grouped by maternal dietary iron intake during pregnancy. **(D)** Canonical correspondence analysis (CCA) conducted between maternal dietary iron intake and the composition of the neonate gut microbiota at the genus level. Neonate gut microbiota genera were assessed as response data and maternal dietary iron intake was explanatory data. Samples with purple, green, blue, and red were denoted by “VD_Higher iron,” “VD_Lower iron,” “CS_Higher iron,” “CS_Lower iron,” groups, respectively. **(E)** Variation explained by maternal dietary iron intake was 10.24%, *p* = 0.007. *Means *p* < 0.05.

The core genera were identified with a threshold of 0.01% in 95% of maternal fecal samples, and a threshold of 0.01% in 75% of neonate fecal samples. In maternal fecal samples, there were seven core genera dominated by *Bifidobacterium*, then *Escherichia* and *Blautia* (Figure 2A and Table 2), with no significant differences between the maternal higher iron and lower iron groups. While in neonate fecal samples stratified by delivery mode, there were eight core genera in NVD dominated by the top three *Escherichia*, *Bifidobacterium*, and *Streptococcus* (Figure 2B and Table 2), and seven core genera in CS dominated by the top three *Enterococcus*, *Escherichia*, and *Staphylococcus* (Figure 2C and Table 2). We identified a higher relative abundance of *Bifidobacterium* in NVD and CS in the higher group of maternal dietary iron intake than that in the lower group, but the differences were not statistically different (*p* = 0.177; *p* = 0.157, respectively). No significant difference was observed in the remaining core genera in groups.

**TABLE 2:** Relative abundance of core genus of mother and neonate samples grouped by maternal dietary iron intake during pregnancy, median (P25–P75).

Core genus	General	Higher iron	Lower iron
**Mother**			
*Bifidobacterium*	31.1 (15.6–52.3)	25.8 (15.5–53.2)	33.2 (19–50.9)
*Escherichia*	14 (4.1–35.7)	13.4 (4.6–33.8)	14 (4.1–38.7)
*Blautia*	13.4 (6.6–21.6)	13.6 (6.4–20)	13.1 (8.2–25.5)
*Subdoligranulum*	7.6 (2.5–17.2)	7.1 (4.7–22.7)	9.3 (2.2–13.6)
*Fusicatenibacter*	3.5 (1.3–7.6)	3.8 (2.2–7.6)	3.5 (1–7.3)
*Clostridium*	2.2 (0.7–4.7)	2.3 (1–5.6)	2 (0.4–3.7)
*Dorea*	2 (1–4.2)	1.8 (1.1–3.6)	2.5 (1–4.6)
**Neonate**			
**NVD**			
*Escherichia*	34.2 (14.7, 82.3)	27.4 (11.7, 57.7)	37.7 (18.9, 89.3)
*Bifidobacterium*	12.3 (3.5, 28.2)	17.2 (3.6, 37.7)	10.3 (1.8, 26.7)
*Streptococcus*	2.3 (0.4, 10.9)	2.8 (0.5, 13)	2.3 (0.3, 7.4)
*Enterococcus*	1.3 (0, 7.7)	1.6 (0, 6.8)	1 (0, 7.9)
*Staphylococcus*	0.2 (0, 3.4)	0.2 (0, 3.3)	0.2 (0, 3.6)
*Roseburia*	1.2 (0, 5.8)	1.3 (0.1, 5)	0.9 (0, 6.4)
*Romboutsia*	0.3 (0, 2.5)	0.2 (0, 2.1)	0.5 (0, 2.6)
*Blautia*	0.5 (0.1, 4.2)	0.6 (0.1, 4.1)	0.4 (0.1, 4.3)
**CS**			
*Enterococcus*	12.6 (3.8, 63.5)	13.4 (4.2, 46.4)	10.7 (3.8, 76.5)
*Escherichia*	11.6 (1.6, 37)	26.7 (5.1, 41.5)	5.3 (1.6, 21.1)
*Staphylococcus*	0.9 (0, 5.7)	1.4 (0, 18.3)	0.8 (0.5, 1.5)
*Streptococcus*	6.3 (1, 13.1)	10.3 (3.1, 14.7)	3.3 (0.8, 10.8)
*Klebsiella*	1.8 (0.2, 4.9)	2.6 (0.4, 5.5)	0.3 (0, 4.8)
*Bifidobacterium*	0.8 (0.3, 6.5)	4.4 (0.4, 21.9)	0.5 (0.2, 5.3)
*Blautia*	0.4 (0.1, 7.1)	2.6 (0.2, 9)	0.2 (0, 0.5)

There were five shared ASVs between mothers and NVD neonates and eight shared ASVs between mothers and CS neonates in the higher iron group. While there were four shared ASVs between mothers and NVD neonates and two shared ASVs between mothers and CS neonates in the lower iron group, as described in Supplementary Table 1. The dominant shared species between mother and NVD neonate in higher group were E. coli (ASV1), B. pseudocatenulatum (ASV2), B. longum (ASV3), S. salivarius (ASV7) and R. faecis (ASV10), while between mother and CS neonate in higher group were E. coli (ASV1), B. pseudocatenulatum (ASV2), S. salivarius (ASV7), B. adolescentis (ASV8), B. obeum (ASV9), R. faecis (ASV10), F. prausnitzii (ASV17) and F. prausnitzii (ASV21). Meanwhile, the shared species between mother and NVD neonates in lower group were E. coli (ASV1), B. pseudocatenulatum (ASV2), B. longum (ASV3) and B. adolescentis (ASV8), while between mother and CS neonates in lower group were E. coli (ASV1) and B. pseudocatenulatum (ASV2).

### 3.5 General attributes of maternal dietary iron intake during pregnancy with respect to neonate fecal microbiota

CCA was conducted at the genus level, as shown in Figure 2D. Genera including *Staphylococcus*, *Faecalibacterium*, *Lactobacillus*, and *Bifidobacterium* were positively correlated with maternal dietary iron intake. Among them, *Staphylococcus* displayed the strongest correlation, while *Lactobacillus* and *Bifidobacterium* showed comparatively weaker correlations. Klebsiella demonstrated a negative correlation with maternal dietary iron intake and was more prevalent in the lower iron group in CS neonates. Furthermore, maternal dietary iron intake was significantly associated with the neonate GM composition; variation explained 10.24% with statistical evidence (*p* = 0.007, Figure 2E).

### 3.6 Effects of maternal dietary iron intake on neonate GM composition and structure

**FIGURE 3:**
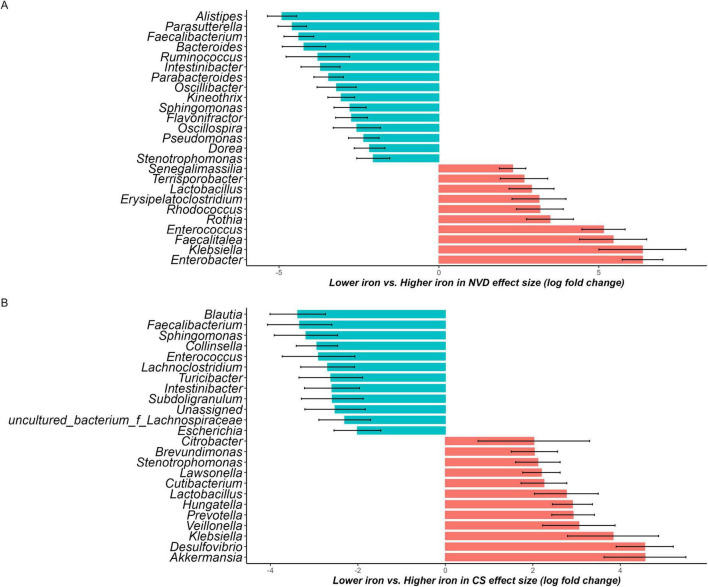
Waterfall plot of differentially abundant genus in the neonate microbiota derived from the ANCOM-BC model, representing beta values (log fold change) by **(A)** normal vaginal delivery (NVD) and **(B)** cesarean section (CS) after birth. *X*-axis represents log fold change of beta values in differential abundance of taxa in Lower iron group versus Higher iron group, while *Y*-axis represents differentially abundant taxa at genus level. All effect sizes were adjusted by Bonferroni method (*q* < 0.05). Taxa with proportion of zeroes greater than 90% was excluded. Taxa represented by blue bars are abundant in Lower iron group, while those represented by red bars are abundant in Higher iron group. Genera with a coefficient greater than 2 were selected for visualization to enhance clarity (the complete list is available in Supplementary Table 2).

In the NVD group there were 35 genera shown to be significantly different between the lower and higher maternal dietary iron intake group, after adjustment for covariates. There were 21 differential genera in the lower group with the most dominant *Alistipes* (beta = −4.91, w = −10.8, *q* < 0.001), *Parasutterella* (beta = −4.59, w = −10.27, *q* < 0.001), and *Faecalibacterium* (beta = −4.38, w = −9.4, *q* < 0.001), respectively. There were 14 differential genera in the higher iron group, where *Enterobacter* (beta = 6.37, w = 10.01, *q* < 0.001) dominated. In the CS group there were 45 genera shown to be significantly different between lower and higher groups, after adjustment for covariates. There were 18 differential genera in the lower group and 27 differential genera in the higher group (Supplementary Table 2). Genera with a coefficient greater than 2 were selected for visualization to enhance clarity in Figure 3. The abundance of *Lactobacillus* was differential in the higher group for both NVD and CS (beta = 2.9, w = 4.13; beta = 2.8, w = 3.8), respectively.

## 4 Discussion

Our results revealed that in rural areas, maternal dietary iron intake during pregnancy is associated with neonate GM alpha diversity, especially for Simpson index before and after stratification by delivery mode. It also positively influences the relative abundance of core genera such as *Bifidobacterium* in the neonatal GM, and was demonstrated association with the neonate GM composition.

The study found that neonates of mothers with relatively higher iron intake within the observed interquartile range (16.9–21.0 mg/day) had greater gut microbiome alpha diversity, particularly in Simpson diversity. Theoretically, higher iron can provide essential nutrients for microbial growth and colonization, and potentially lead to higher alpha diversity of neonatal GM, the presence of other microbial species except *Lactobacillus* and *Bifidobacterium*, albeit in lower abundance, can contribute to the overall ecosystem stability and functionality with complementary benefits (31). Promoting greater diversity and stability within the neonatal gut microbiota is a critical area needing further exploration. Increased microbial heterogeneity has been consistently associated with enhanced metabolic function, immune system development, and resistance to pathogenic colonization during this vulnerable stage of life. However, the specific relationship between the diversity of the early-life gut microbiota and its downstream effects on colonization patterns and functional capacities requires further investigation.

In neonatal GM, the “core genus” represents microbial taxa essential for early colonization and immune development (32, 33). VanOrmer et al. (34) found that in breastfed preterm neonates whose mother received iron supplementation, no significant differences were found in either phyla or key genera relative abundance between pre- and post-iron timepoints. We observed similar results in full-term infants, where maternal dietary iron intake within appropriate limits, whether high or low, did not affect the abundance of core genera, the underlying reasons and mechanisms require further study. *Bifidobacterium* plays a critical role in immune system maturation and gut microecological balance (35, 36), but the association between iron and *Bifidobacterium* in the neonatal gut remains inconsistent. While moderate dietary iron may promote the colonization of beneficial microbiota (37), iron-fortified diets have been shown to decrease *Bifidobacterium* and *Lactobacillus* levels while increasing pathogenic *Escherichia coli*, which is linked to enteritis (38–40). This underscores the importance of rational dietary iron intake for microbiota health. Additionally, we observed that *Lactobacillus* species were enriched in higher iron groups across both delivery modes, suggesting a potential connection between adequate iron intake and *Lactobacillus* presence (41). Previous studies have reported mixed outcomes regarding the impact of probiotics on iron levels. For instance, one study indicated that children receiving iron plus *Lactobacillus reuteri* had higher hemoglobin levels compared to those receiving iron alone. Conversely, another trial found no significant correlation between *Lactobacillus plantarum* and increased ferritin levels. These discrepancies may stem from differences in specific gut strains, yet it is evident that excessive iron intake can reduce beneficial bacteria, including *Lactobacillus* and *Bifidobacterium* (42, 43). Thus, our findings suggest that maternal dietary iron intake within recommended limits may enhance the relative abundance of core genera in neonates, particularly in iron-deficient regions.

Based on the FFQ, the main dietary sources of iron were red meat (mainly pork), dark green leafy vegetables, and legumes, which are common in rural maternal diets. Iron supplements were also recorded but contributed less to total intake. Our study preliminarily evidenced an association between maternal dietary iron intake during pregnancy and neonate GM. While there are recommended dietary iron intake standards for pregnant women, there remain populations who are unable to meet these targets due to economic or social constraints. This study further emphasizes the need to prioritize and support these vulnerable groups. We employed a locally validated FFQ to accurately assess the average dietary intake throughout the entire pregnancy. This approach offers convenience and cost-effectiveness for both investigators and participants. The high-throughput sequencing analysis of fecal samples reflected the impact on the composition of neonatal gut from the perspective of microbiome. However, there are still some limitations. First, to ensure the accuracy of the study, we screened the FFQ and fecal samples, and considering the potential impact on the composition of GM, we then stratified the groups by delivery mode, causing the limited sample size especially in CS stratification, and making it difficult to accurately reflect the more subtle differences. Additionally, we were not able to analyze the dose-response relationship between iron and gut microbiota characteristics due to limited sample size. As an exploratory study, we plan to expand our study by including a larger number of participants to strengthen the statistical results and improve the generalizability of our findings in the future research. Second, unfortunately, we did not collect indicators related to iron metabolism, so we cannot clarify how dietary iron intake affects iron metabolism and, consequently, the function of the infant gut microbiota. We plan to include iron metabolism indicators as important variables in the future study. Third, rarefaction is still a topic of debate in academia, we concerned about the low biomass of neonate gut microbiota, so we filtered the ASV rather than rarefaction. We will follow this progress in future studies in order to adopt the best way to analyze microbiota data. Additionally, there may still be unmeasured or unknown variables that may influence the neonatal GM, such as maternal enterotypes, which were not assessed in this study but could potentially affect the vertical transmission of microbiota. The inclusion of specific confounding factors may be relevant to the specific population and study settings. Thus, future studies could consider combining multiple type of biological samples to assess iron influence and metabolism *in vivo* within the framework of a larger sample size and generalizing the results to more regions.

## 5 Conclusion

Adequate and within appropriate limits iron intake during pregnancy may promote the colonization of beneficial bacteria in the neonate GM and increase its biodiversity, indicating that maternal dietary iron intake plays a role in shaping the GM of the neonate, especially in developing areas with low dietary iron intake. Different dietary intervention policies should be adopted to ensure adequate intake of iron during pregnancy to promote healthy GM development in neonates.

## Data Availability

The data presented in the study are deposited in the NCBI Sequence Read Archive (SRA), accession number PRJNA1278969.

## Supplementary material

Supplementary Figure 1 The flowchart of participant enrolment.

Supplementary Figure 2 The dominant phylum composition and top phyla of mother and neonate samples. Mother and neonate samples were grouped by maternal dietary iron intake into higher iron and lower iron groups. **(A)** The dominant phyla of maternal gut microbiota. **(B,C)** are the top phyla and genera of mother samples. **(D)** The dominant phyla of neonate gut microbiota. **(E,F)** are the top phyla and genera of neonate samples. The comparison between groups was analyzed by non-parametric test, and there was no significant difference between two groups (*p* > 0.05).

Supplementary Figure 3 The dominant phylum composition, top phyla, and top genera of neonate samples. The neonate samples were stratified by delivery mode and grouped by maternal dietary iron intake. **(A)** The dominant phyla of vaginal delivered neonate gut microbiota. **(B)** The top phyla of neonate samples. **(C)** The dominant phyla of caesarean section delivered neonate gut microbiota. **(D)** The top genera of neonate samples. The comparison between groups was analyzed by non-parametric test, and there was no significant difference between higher iron and lower iron groups (*p* > 0.05).

Supplementary Figure 4 Alpha, beta diversity indexes of the maternal gut microbiota grouped by maternal dietary iron intake into higher iron and lower iron groups. **(A)** Chao1 index regarding the microbial community richness of the maternal gut microbiota; Shannon **(B)** and Simpson **(C)** index regarding the microbial community diversity of the maternal gut microbiota; Each box plot represents the median, interquartile range, minimum, and maximum values. **(D)** PCoA analysis of bray_curtis distance regarding the difference in the microbial community composition (PERMANOVA with 9999 permutations, *p* > 0.05).
