# Supplementary material for: Maternal dietary iron intake during pregnancy has a potential effect on the neonate gut microbiota profile
Source: Front Nutr. 2025 Jun 24;12:1589258. doi: 10.3389/fnut.2025.1589258 (PMC12234286; doi:10.3389/fnut.2025.1589258)
Supplement: Supplementary Figure 1 — The flowchart of participant enrolment. [file Supplementary_file_1.zip › Supplementary figures.docx]

**Supplementary Figures**

**Supplementary Figure 1.** The flowchart of participant enrolment

**Supplementary Figure 2.** The dominant phylum composition and top phyla of mother and neonate samples.

**Supplementary Figure 3.** The dominant phylum composition, top phyla, and top genera of neonate samples.

**Supplementary Figure 4.** Alpha, beta diversity indexes of the maternal gut microbiota grouped by maternal dietary iron intake into higher iron and lower iron groups.

The entire captions are below.


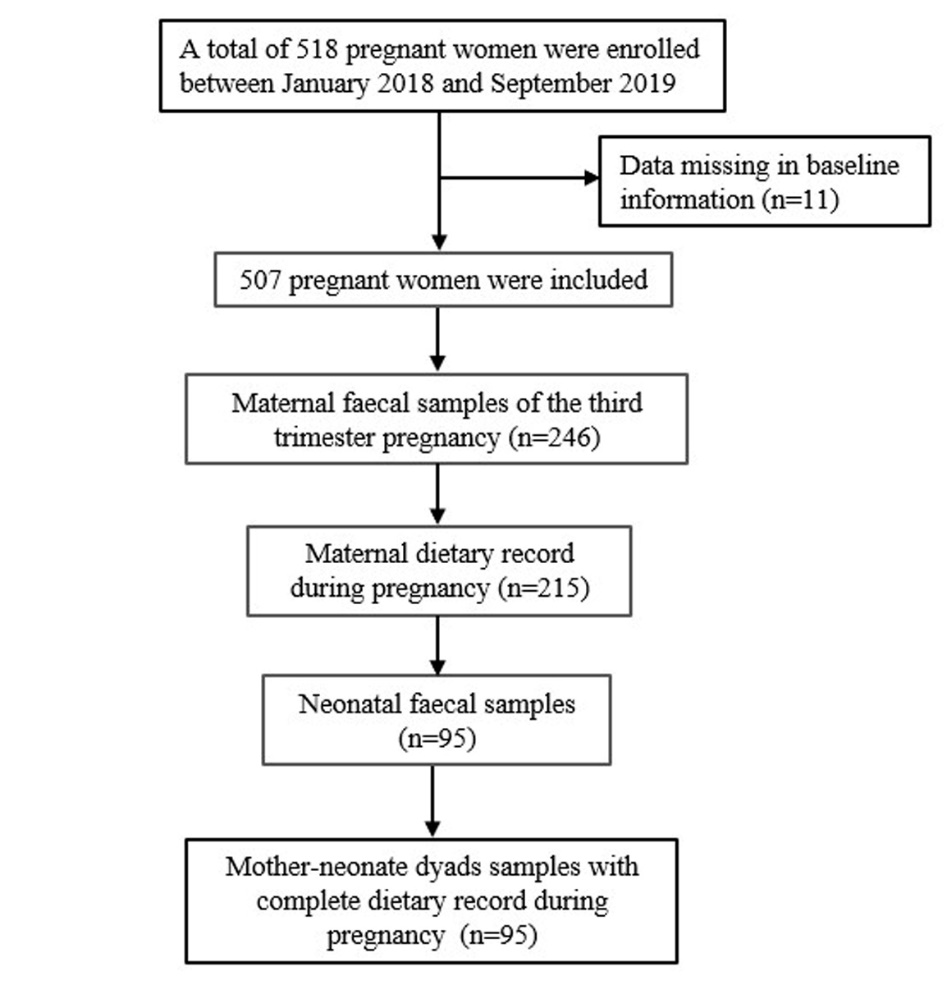


**Supplementary Figure 1.** The flowchart of participant enrolment

**
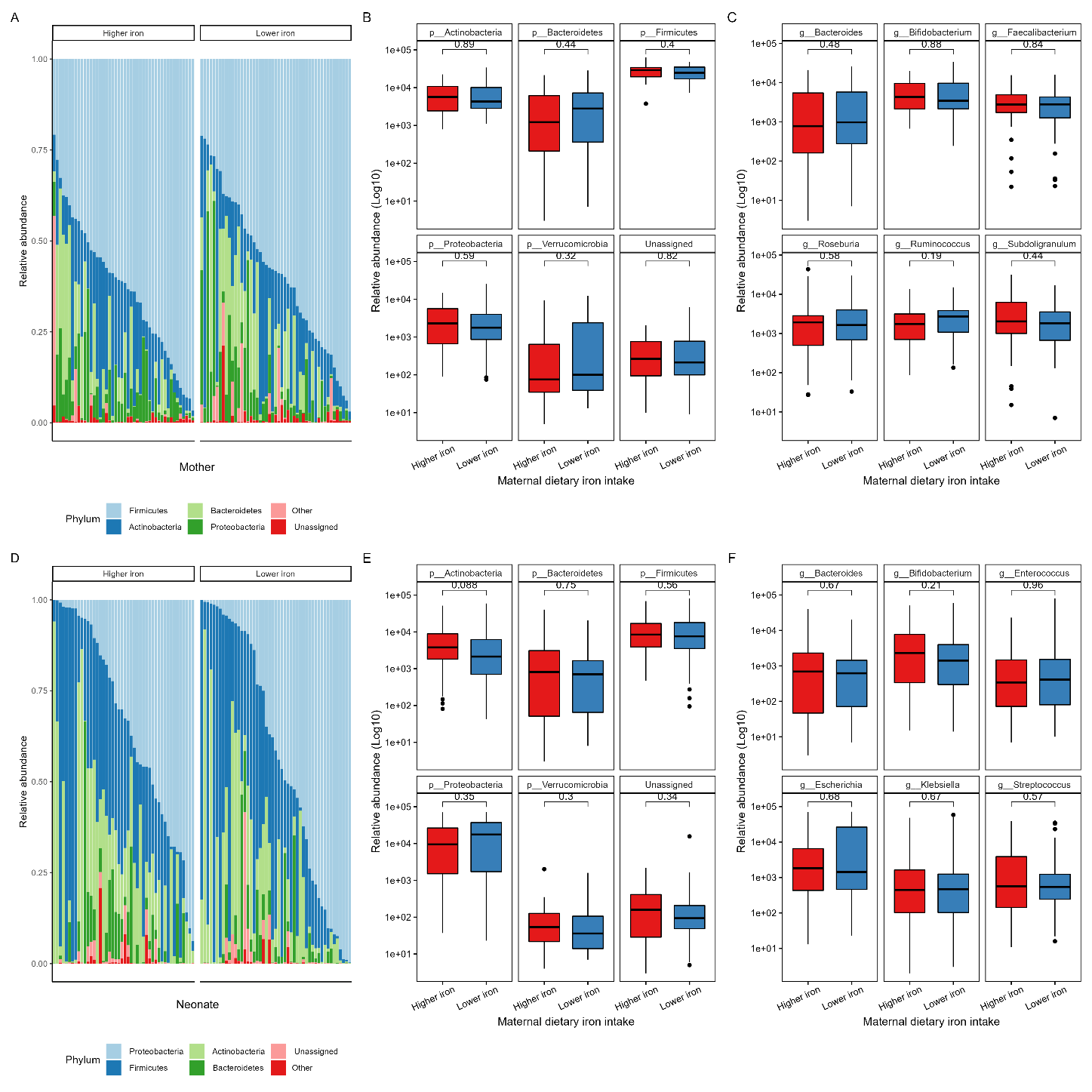
**

**Supplementary Figure 2.** The dominant phylum composition and top phyla of mother and neonate samples. Mother and neonate samples were grouped by maternal dietary iron intake into higher iron and lower iron groups. (A) The dominant phyla of maternal gut microbiota. (B) and (C) are the top phyla and genera of mother samples. (D) The dominant phyla of neonate gut microbiota. (E) and (F) are the top phyla and genera of neonate samples. The comparison between groups was analysed by non-parametric test, and there was no significant difference between two groups (*p* >0.05).


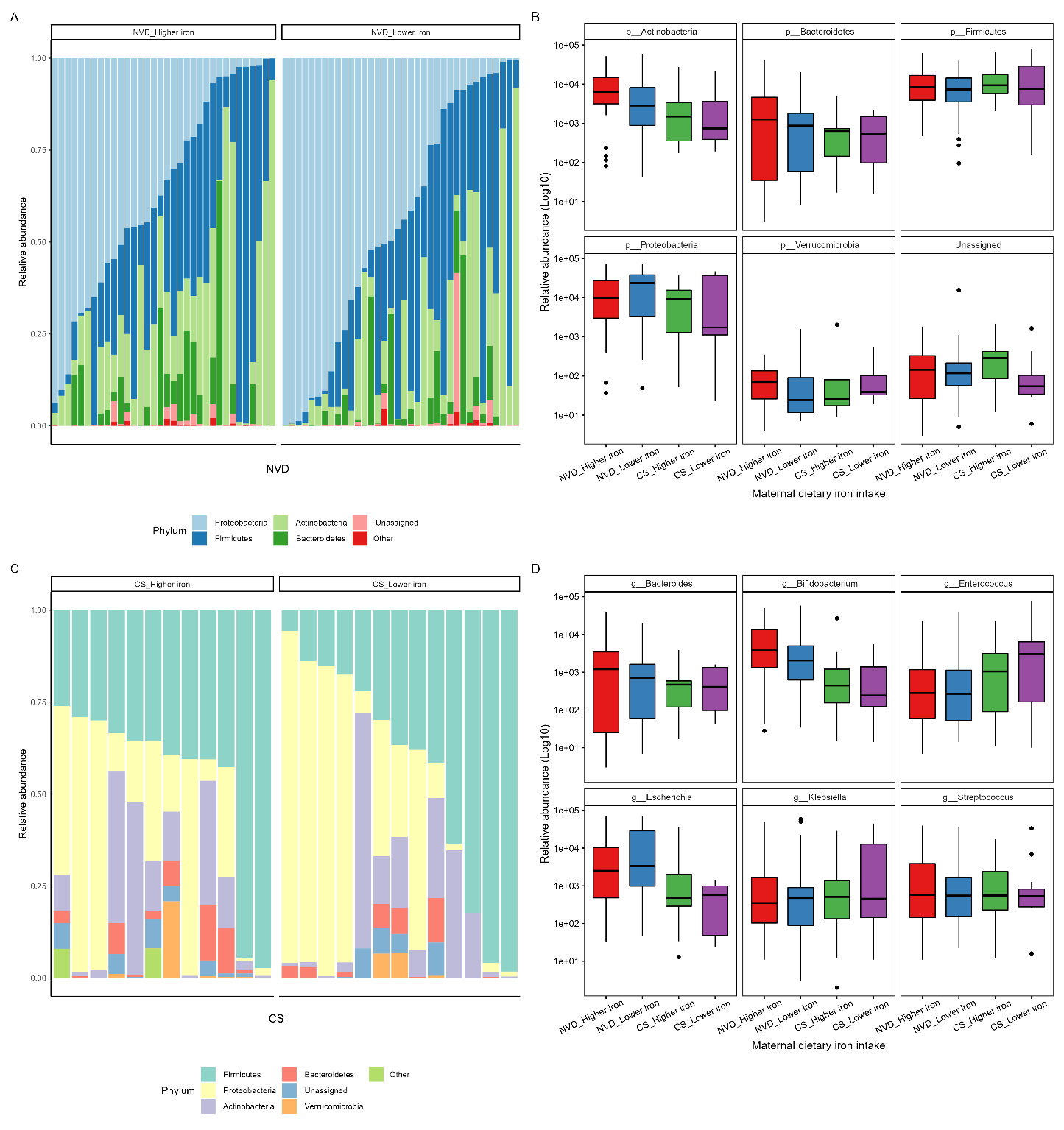


**Supplementary Figure 3.** The dominant phylum composition, top phyla, and top genera of neonate samples. The neonate samples were stratified by delivery mode and grouped by maternal dietary iron intake. (A) The dominant phyla of vaginal delivered neonate gut microbiota. (B) The top phyla of neonate samples. (C) The dominant phyla of caesarean section delivered neonate gut microbiota. (D) The top genera of neonate samples. The comparison between groups was analysed by non-parametric test, and there was no significant difference between higher iron and lower iron groups (*p* >0.05).


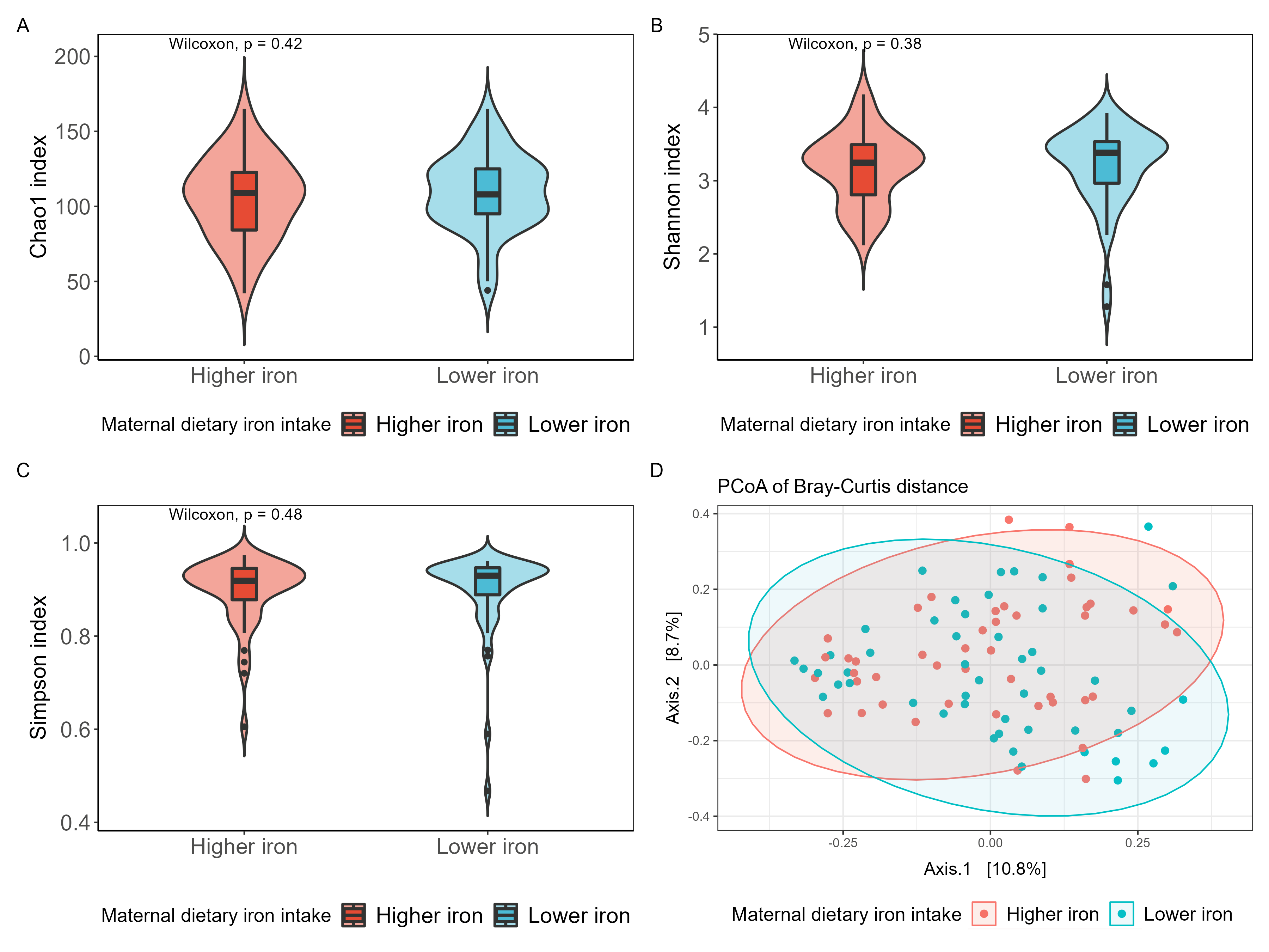


**Supplementary Figure 4.** Alpha, beta diversity indexes of the maternal gut microbiota grouped by maternal dietary iron intake into higher iron and lower iron groups. (A) Chao1 index regarding the microbial community richness of the maternal gut microbiota; Shannon (B) and Simpson (C) index regarding the microbial community diversity of the maternal gut microbiota; Each box plot represents the median, interquartile range, minimum, and maximum values. (D) PCoA analysis of bray_curtis distance regarding the difference in the microbial community composition (PERMANOVA with 9999 permutations, *p* >0.05).
